# Histone methyltransferase NSD2 regulates apoptosis and chemosensitivity in osteosarcoma

**DOI:** 10.1038/s41419-019-1347-1

**Published:** 2019-01-25

**Authors:** Chao He, Chao Liu, Lei Wang, Yangbai Sun, Yuhang Jiang, Yongqiang Hao

**Affiliations:** 1 Shanghai Key Laboratory of Orthopedic Implants, Department of Orthopedic Surgery, Shanghai Ninth People’s Hospital, Shanghai Jiao Tong University School of Medicine, Shanghai, China; 2 Department of Oromaxillofacial Head and Neck Oncology, Shanghai Ninth People’s Hospital, College of Stomatology, Shanghai Jiao Tong University School of Medicine, Shanghai, China; 3 Department of Musculoskeletal Oncology, Fudan University Shanghai Cancer Center, Shanghai Medical College, Fudan University, Shanghai, China

## Abstract

Osteosarcoma (OS) is a primary malignant bone tumour. However, the genetic basis for the pathogenesis of OS remains elusive. In this study, we uncovered the role of the histone methyltransferase NSD2 in regulating tumourigenesis and chemosensitivity in OS. We show that NSD2 knockdown leads to increased apoptosis in OS cells in vitro and in vivo. Additionally, NSD2 knockdown significantly enhances the efficacy of cisplatin against OS cells and accordingly inhibits properties associated with cancer stem cells (CSCs). Furthermore, RNA sequencing (RNAseq) and Gene Ontology (GO) analysis revealed that NSD2 promotes transcription of genes associated with negative regulation of apoptotic signalling pathways and CSC properties. The results of chromatin immunoprecipitation quantitative polymerase chain reaction (ChIP-qPCR) assays indicated that NSD2 knockdown leads to decreased H3K36me2 modification at *BCL2* and *SOX2* loci, thus inhibiting the transcription of these two genes that are closely correlated with apoptosis, CSC properties and chemosensitivity in OS cells. Pathway analysis demonstrated that the ERK and AKT pathways mediate the regulation of OS progression and chemosensitivity by NSD2. Overall, our study is the first to uncover the function of NSD2 in OS chemosensitivity. NSD2 regulates the expression of the apoptosis regulatory proteins BCL2 and SOX2 through the ERK and AKT pathways. Our results suggest that NSD2 is a new target for combined chemotherapy and is a prognostic factor for OS.

## Introduction

Osteosarcoma (OS) is one of the most common malignant bone tumours and arises primarily in children and adolescents^1^. In adults aged >65 years, OS develops as a secondary malignancy related to Paget’s disease of bone^2^. To date, chemotherapy has been frequently included in conventional treatment of OS along with surgical resection. Since the 1970s, the introduction of adjuvant chemotherapies to OS treatment has impressively improved the 5-year survival rate. In addition, in patients with localized disease, the strongest predictor of overall survival is the patient’s response to preoperative combination chemotherapy^3^. Unfortunately, the 5-year survival rate has remained at approximately 20% over the past 20 years without changing^4,5^, which is partly due to chemoresistance. Cisplatin is a National Comprehensive Cancer Network first-line chemotherapy medication commonly used for clinical treatment of OS and a variety of other tumours^3^. In OS patients, cisplatin shows a response rate of approximately 30%, indicating that a significant proportion of OS patients are intrinsically resistant to cisplatin. Therefore, there is an urgent need to develop new strategies to further improve the long-term survival rates of OS patients.

Epigenetic perturbations caused by histone methyltransferases or demethylases are recognized as important contributing factors to a variety of tumours^6^, and epigenetic markers have frequently been found to be mutated or dysregulated in multiple cancers^7^. NSD2, which belongs to the NSD family of histone lysine methyltransferases (HMTases)^8^, is a histone methyltransferase that mediates dimethylation of histone 3 lysine 36 (H3K36me2); H3K36 methylation is typically a permissive marker associated with transcriptional activation^9–11^. NSD2 was first reported to function as an oncogenic gene in multiple myeloma^12–14^. In addition, NSD2 is highly expressed in many solid tumours, such as those in prostate cancer, breast cancer and head and neck cancer^12–14^. However, whether NSD2 mediates chemosensitivity in OS has not been reported.

In this study, the results indicated that NSD2 is upregulated in OS tissues compared with normal tissues and that NSD2 knockdown can enhance OS apoptosis and sensitize OS to cisplatin by directly decreasing H3K36me2 levels at *BCL2* and *SOX2* gene loci, as measured by chromatin immunoprecipitation (ChIP) analysis. Pathway analysis illustrated that the extracellular signal–regulated kinase (ERK) and AKT signalling pathways are reversed when NSD2 is knocked down. Together, our results suggest that NSD2 is a novel target for overcoming chemoresistance in OS.

## Results

### NSD2 expression is upregulated in OS and in cisplatin-resistant patients

**Fig. 1:**
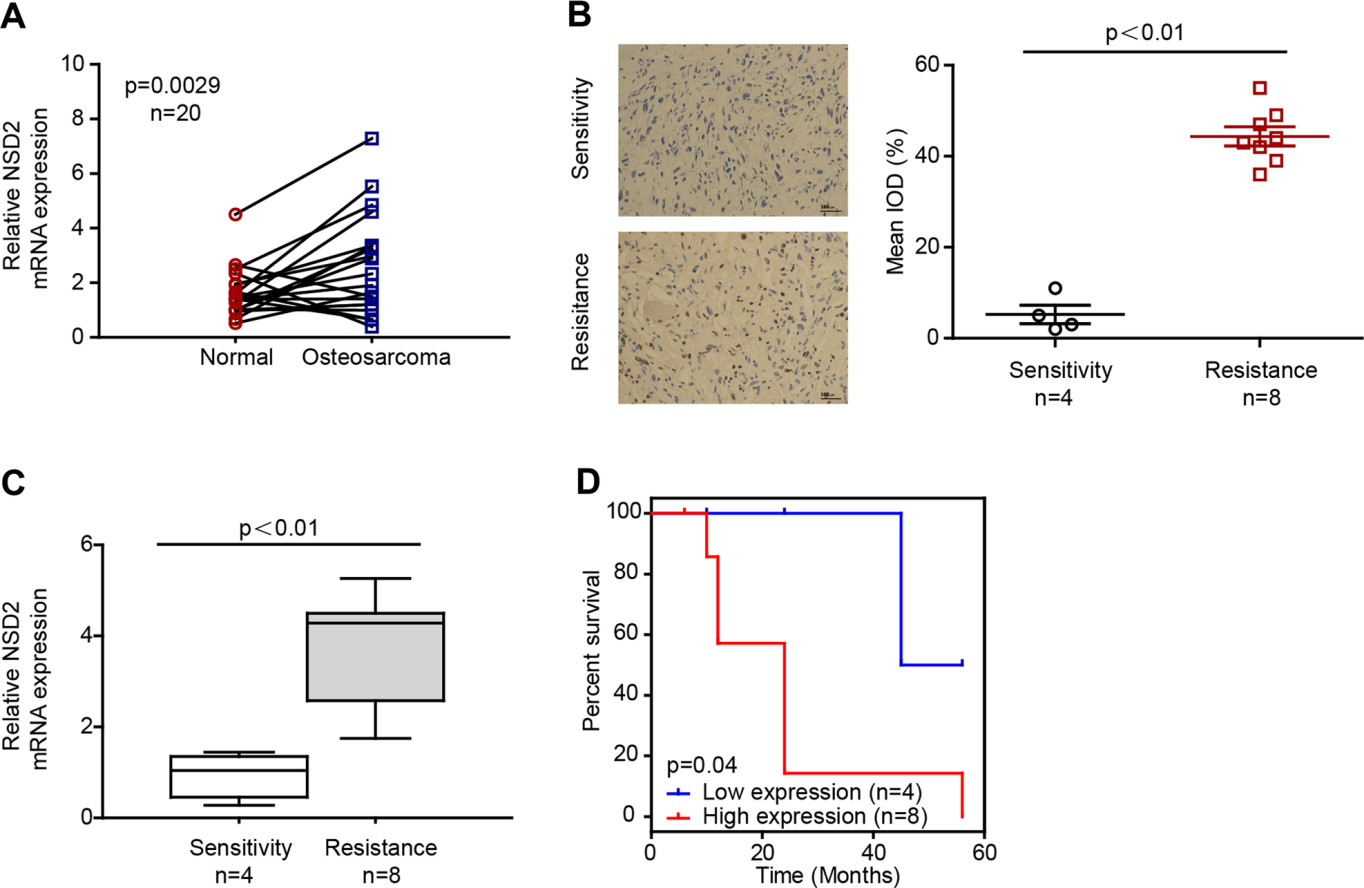
NSD2 expression is upregulated in osteosarcoma and in cisplatin-resistant patients. **a** NSD2 mRNA levels in 20 tumoural and matched peritumoural tissues. NSD2 expression in cisplatin-sensitive and cisplatin-resistant specimens was measured by immunohistochemistry (**b**) and real-time quantitative polymerase chain reaction (**c**). **d** Kaplan–Meier plot of survival time in patients with low and high NSD2 expression. IOD integrated optical density

To assess the role and clinical relevance of NSD2 in OS, we first assessed NSD2 expression in OS and matched normal peritumoural specimens. Real-time quantitative polymerase chain reaction (RT-qPCR) analysis showed that NSD2 mRNA expression was significantly higher in OS tissues than in matched normal tissues in 20 OS patient biopsies (Fig. 1a). The clinical information of the 20 OS patients is summarized in Table S1. Furthermore, NSD2 expression was higher in cisplatin-resistant OS biopsies than in cisplatin-sensitive ones, as measured by immunohistochemistry (IHC) and RT-qPCR (Fig. 1b, c). Patients with lower NSD2 expression presented better prognoses (Fig. 1d); the relative clinical information is listed in Table S2. Together, these findings suggest that NSD2 is upregulated in OS patients, especially in cisplatin-resistant OS patients, and that lower NSD2 expression predicts better prognoses in OS patients.

### NSD2 knockdown promotes OS apoptosis in vitro

**Fig. 2:**
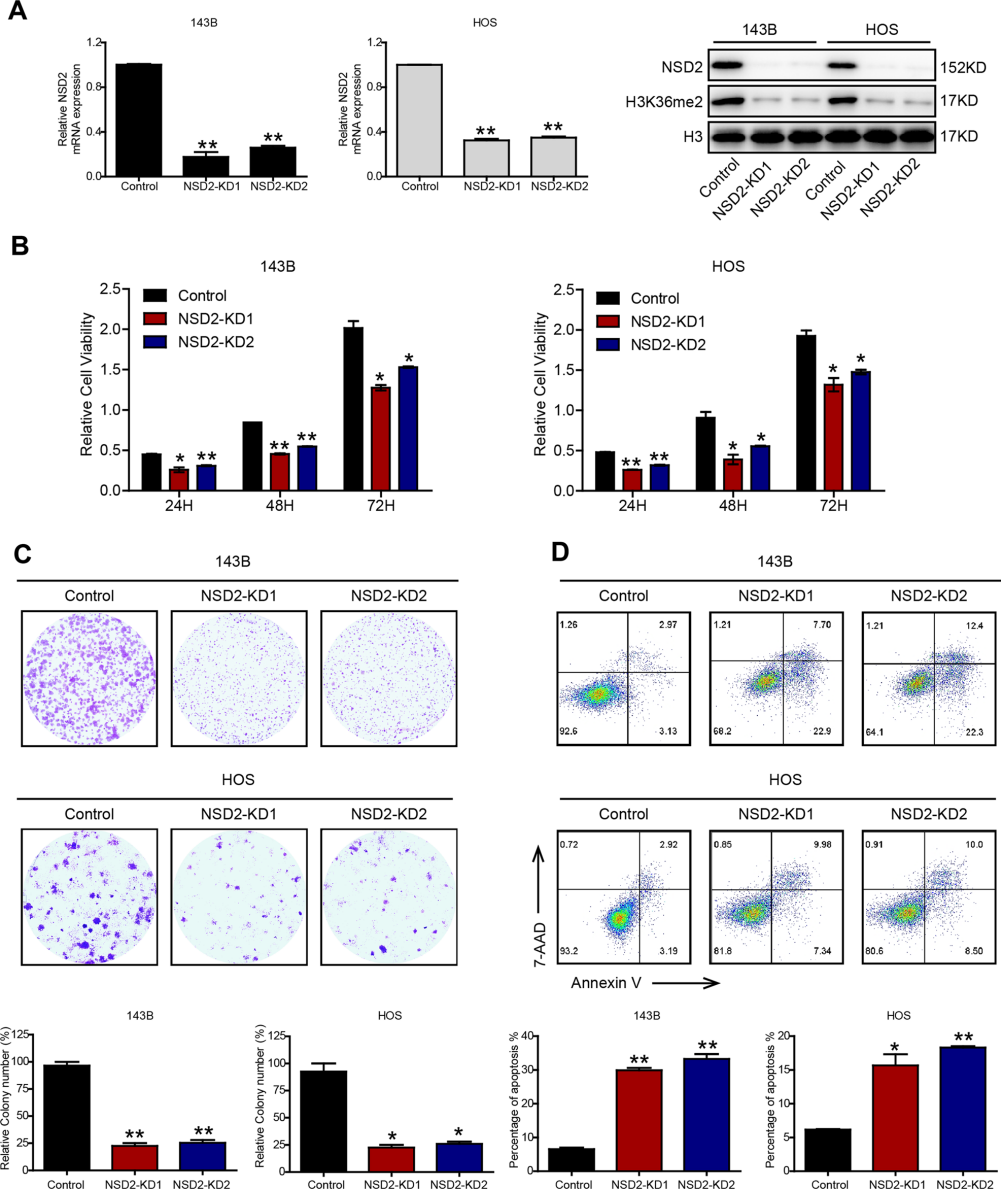
NSD2 knockdown promotes osteosarcoma cell apoptosis in vitro. **a** Real-time quantitative polymerase chain reaction and western blot analysis of the indicated proteins in control and NSD2-KD cells. In vitro growth of control and NSD2-KD 143B and HOS cells was assessed by Cell Counting Kit-8 assay (**b**) and colony-formation assay (**c**). **d** Flow cytometric analysis of apoptosis in control and NSD2-KD OS cells. *P < 0.05; **P < 0.01 compared with the control group

To further test the role of NSD2 in OS, we knocked down NSD2 (NSD2-KD) with a lentiviral system. We generated NSD2-depleted 143B and HOS cells using packaged lentiviruses including two short hairpin RNA (shRNA) sequences targeting NSD2 (the sequences are listed in Table S4) that displayed similar knockdown efficiencies (Fig. 2a). Although previous research on myeloma has indicated that NSD2 can affect the enhancer of zeste homologue 2 (EZH2, a methyltransferase), increase histone 3 lysine 27 trimethylation (H3K27me3) levels and ultimately lead to repression of gene expression^15,16^, the results in our study indicated that H3K27me3 levels were not changed significantly by knockdown of NSD2 (Fig. S1) which is in accordance with the results of Kuo et al.^9^. Moreover, the results also indicated that NSD2 knockdown reduced the growth of OS cells, as measured by Cell Counting Kit-8 (CCK-8) and colony-formation assays (Fig. 2b, c). Notably, the outcomes of flow cytometric analysis indicated that there were no significant differences in cell cycle progression between control and NSD2-KD OS cells (Fig. S2). In addition, the percentage of cells that underwent apoptosis was higher among NSD2-KD OS cells than among parental cells (Fig. 2d). Taken together, our results indicate that NSD2 promotes tumourigenesis and inhibits apoptosis in OS.

### NSD2 knockdown inhibits OS genesis in vivo

**Fig. 3:**
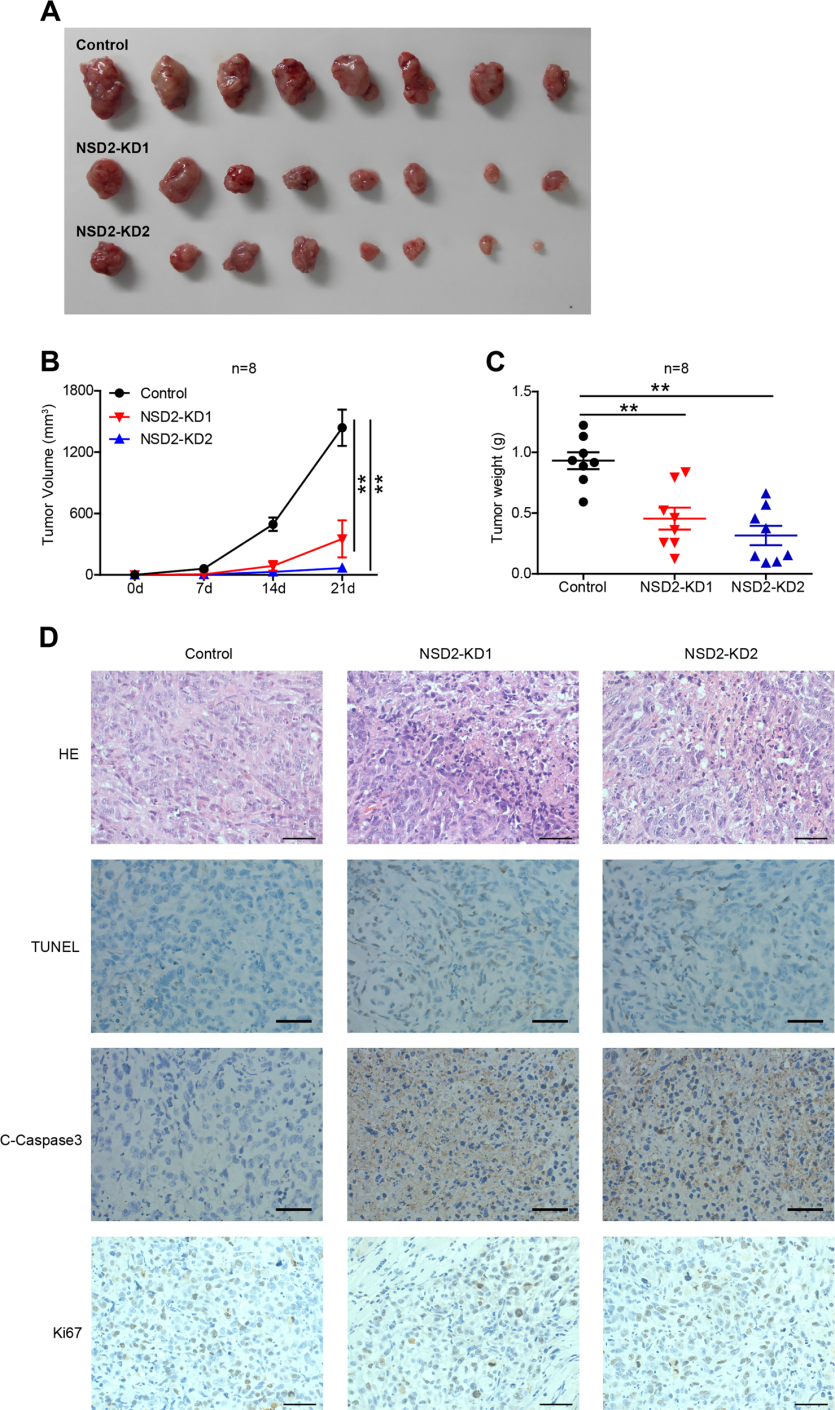
NSD2 knockdown inhibits osteosarcoma genesis in vivo. **a** Macroscopic image of tumour size. Diagrams of tumour volume (**b**) and tumour weight (**c**) for mice sacrificed at the indicated time points are shown. **d** Haematoxylin–eosin staining and transferase-mediated deoxyuridine triphosphate-biotin nick end labelling, cleaved caspase 3 and Ki67 staining of representative tumours in the control group and two NSD2-KD groups. **P < 0.01. Scale bar = 50 μm

Next, we used a xenograft mouse model to determine the role of NSD2 in OS tumourigenesis. Three groups of 6–8-week-old athymic nude mice were subcutaneously injected with control or NSD2-KD 143B cells. Tumour volume was monitored every 7 days, and tumour weight was measured on day 21 when the mice were sacrificed. The data indicated that there were dramatic reductions in the size and weight of OS tumours in the NSD2-KD groups compared with the control group (Fig. 3a–c). Then we assessed a range of markers related to tumour cell apoptosis and proliferation via IHC, the results of which indicated that the numbers of cleaved caspase 3-positive and transferase-mediated deoxyuridine triphosphate-biotin nick end labelling (TUNEL)-positive tumour cells were significantly higher in the two NSD2-KD groups than in the control group, although there were no significant differences in Ki67 expression among the groups (Fig. 3d). Taken together, these data support and verify the observations made in vitro and demonstrate that NSD2 knockdown suppresses OS tumourigenesis and enhances apoptosis in vivo.

### NSD2 deficiency enhances cisplatin efficacy

**Fig. 4:**
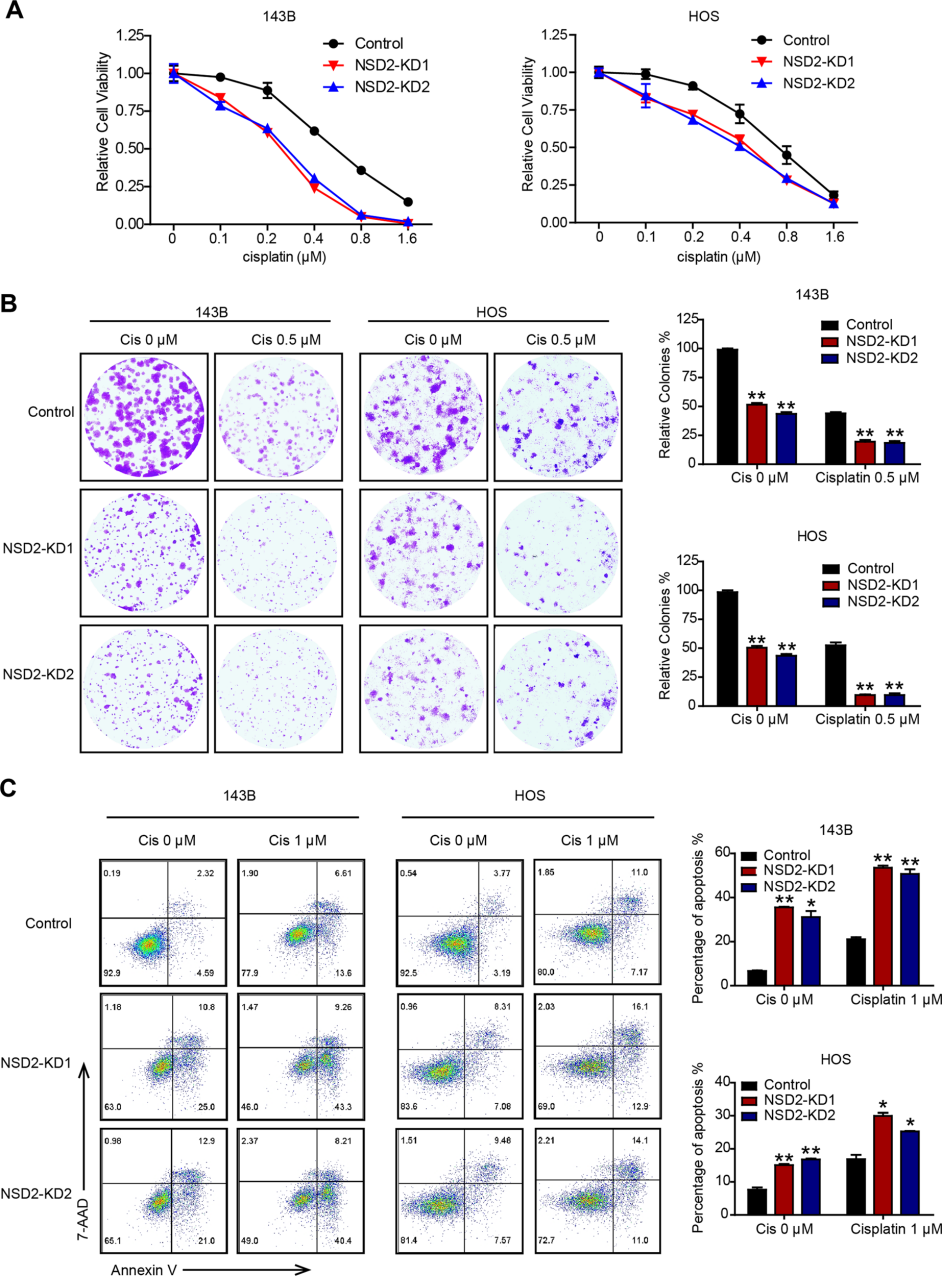
NSD2 deficiency enhances cisplatin efficacy. In vitro growth of control and NSD2-KD 143B and HOS cells with or without cisplatin as assessed by Cell Counting Kit-8 assay (**a**) and colony-formation assay (**b**). **c** Apoptosis in control and NSD2-KD osteosarcoma cells with the indicated treatments was assessed by staining by Annexin V and 7-aminoactinomycin D. *P < 0.05 and **P < 0.01 compared with the control group

Owing to the significant apoptotic enhancement that occurred after NSD2 knockdown in OS cells, we further estimated the synergistic effect of NSD2 on OS chemosensitivity to cisplatin. The results of CCK-8 and colony-formation assays suggested that NSD2 knockdown significantly sensitized OS cells to cisplatin (Fig. 4a, b), and flow cytometric analysis demonstrated that NSD2 deficiency could magnify the cytotoxic effects of cisplatin (Fig. 4c). The percentage of apoptotic cells was approximately two-fold greater among NSD2-KD cells than among control cells after cisplatin (1 μM) treatment. Thus these results indicate that NSD2 knockdown can promote cisplatin efficacy in vitro.

### NSD2 regulates cancer stem cell (CSC) properties in OS

**Fig. 5:**
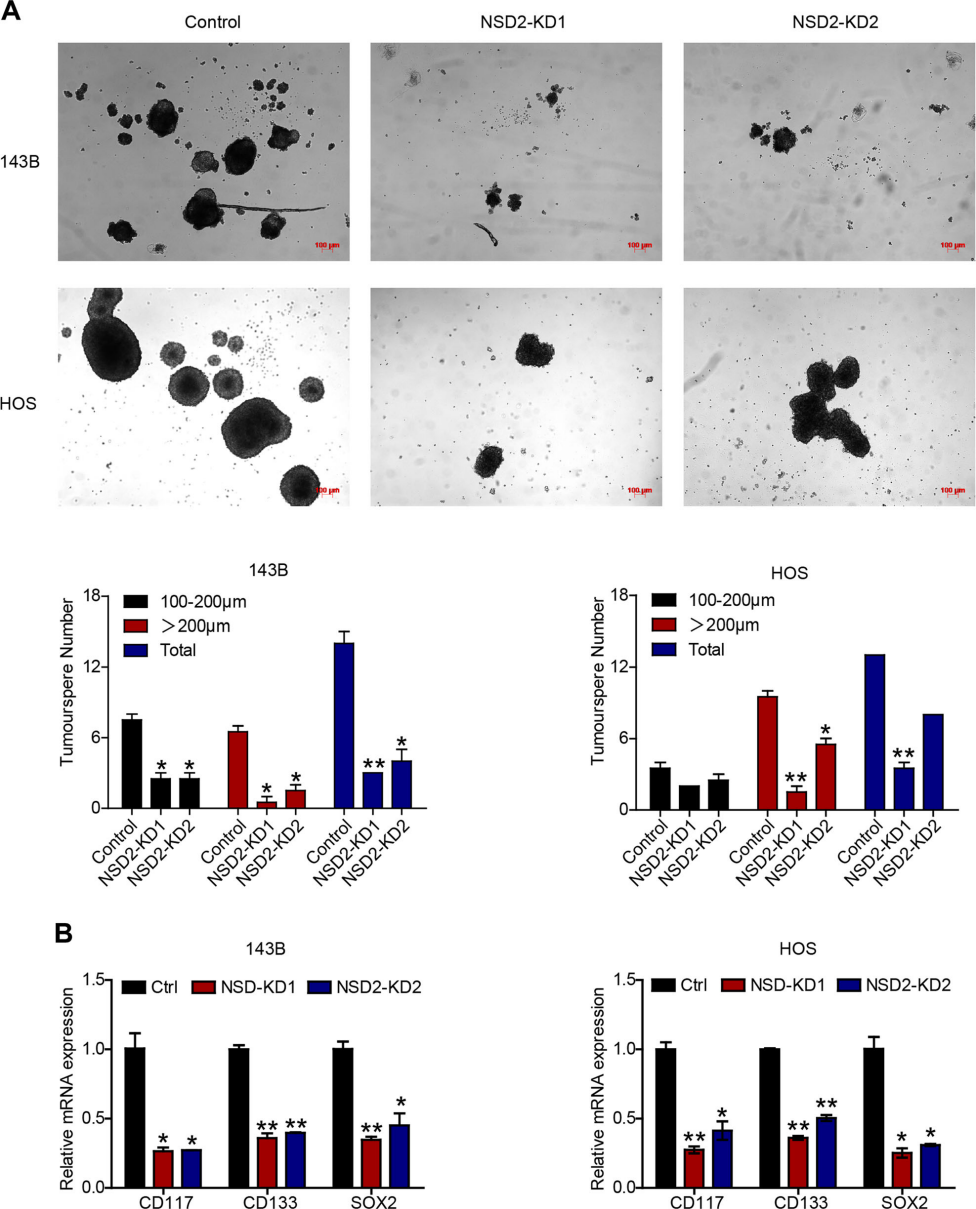
NSD2 regulates cancer stem cell properties in osteosarcoma. **a** Tumoursphere formation in control and NSD2-KD 143B and HOS cells. **b** *CD117*, *CD133* and *SOX2* mRNA expression in parental and NSD2-KD OS cells. *P < 0.05; **P < 0.01 compared with the control group

Accumulating evidence indicates that a specific subset of cells in tumour cell lines or in tumour masses can be classified as CSCs^17^. CSCs are closely correlated with chemoresistance and tumourigenesis in many solid tumours^18,19^. Thus we explored whether NSD2 influences OS CSC properties (stemness). The results of sphere-formation experiments revealed that NSD2-KD decreased the number and size of tumourspheres in OS cells (Fig. 5a), indicating that NSD2 knockdown was associated with reduced CSC properties. Likewise, RT-qPCR also indicated that *CD117*, *CD133* and *SOX2* were repressed in the absence of NSD2 (Fig. 5b). Collectively, these findings highlight that NSD2 promotes CSC properties in OS.

### NSD2 regulates apoptosis- and CSC-related genes

**Fig. 6:**
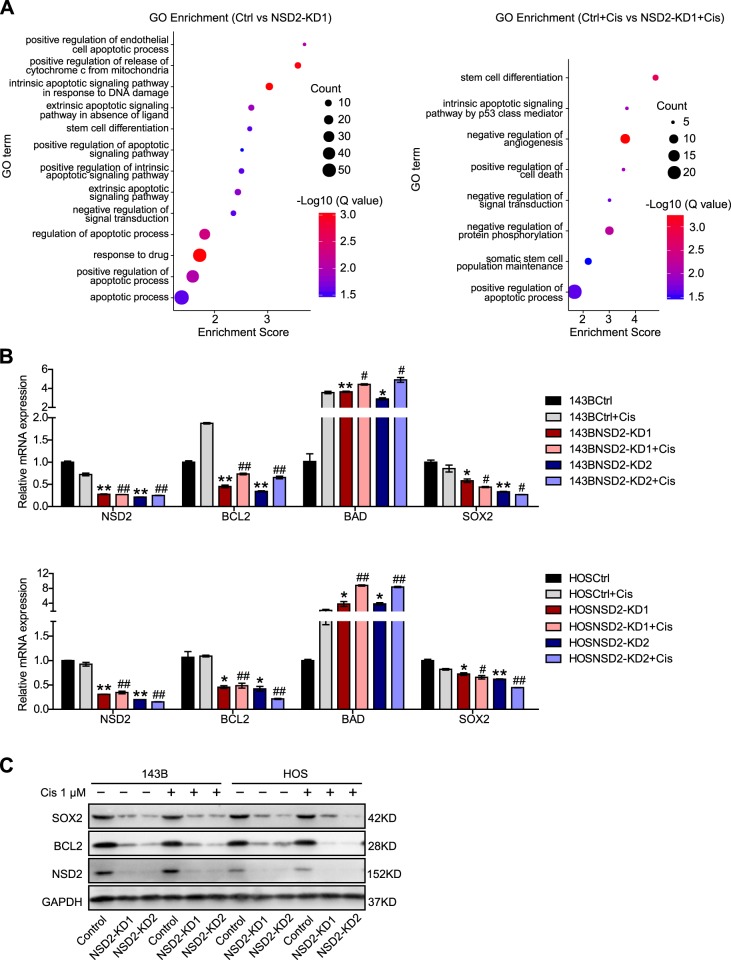
NSD2 regulates apoptosis- and cancer stem cell-related genes. **a** Gene Ontology analysis of differentially expressed genes in NSD2-KD cells compared with control cells without cisplatin (left panel) or with cisplatin (right panel). **b** Real-time quantitative polymerase chain reaction confirmation of *NSD2*, *BCL2*, *BAD* and *SOX2* expression in control and NSD2-KD 143B cells (upper panel) and in HOS cells (lower panel). **c** Western blot analysis of SOX2, BCL2 and NSD2 expression in parental and NSD2-KD OS cells. *P < 0.05 and **P < 0.01 compared with the control group; ^#^*P* < 0.05 and ^##^*P* < 0.01 compared with the cisplatin-treated group

To gain insight into the mechanism by which NSD2 knockdown promotes apoptosis in OS cells and sensitizes the cells to cisplatin, we performed RNA sequencing (RNAseq) using parental and NSD2-KD 143B cells. Gene Ontology (GO) analysis indicated that a number of GO terms associated with cell apoptosis and CSCs, such as “intrinsic apoptotic signalling pathway in response to DNA damage”, “positive regulation of apoptotic signalling pathway”, “response to drug” and “stem cell differentiation”, were enriched for the genes that were differentially expressed in NSD2-KD cells compared with control cells in the presence or absence of cisplatin (Fig. 6a). Based on the results of GO analysis, we then analysed the expression of several genes with RT-qPCR. The results indicated that *BCL2* and Sex-determining region Y-box 2 (*SOX2*) were downregulated but that *BAD* was upregulated when NSD2 was knocked down in OS cells (Fig. 6b). Western blot analysis further verified that with the knockdown of NSD2, BCL2 and SOX2 were downregulated (Fig. 6c). BCL2 and BAD belong to the BCL2 family, which governs the intrinsic apoptosis pathway^20–24^. In general, BCL2 plays an antiapoptotic role, while BAD plays a proapoptotic role. SOX2 belongs to the highly conserved HMG box family of SOX transcription factors and plays essential roles during embryogenesis as a master regulator in pluripotent embryonic stem cells^25^. Several recent studies have shown that exogenous upregulation of SOX2 can promote resistance to chemotherapeutics^26–35^. In addition, higher SOX2 levels have been reported to correlate with poorer prognosis in cancers, such as breast, colorectal, oesophageal, ovarian, prostate, and lung cancer as well as in nasopharyngeal and sinonasal carcinoma^32,33,35–42^. Taken together, these results indicate that NSD2 deficiency downregulates the expression of the antiapoptotic gene *BCL2* and the CSC-associated gene *SOX2* but upregulates the expression of the proapoptotic gene *BAD*.

### NSD2 mediates H3K36me2 modification at *BCL2* and *SOX2* loci and regulates the ERK and AKT signalling pathways

**Fig. 7:**
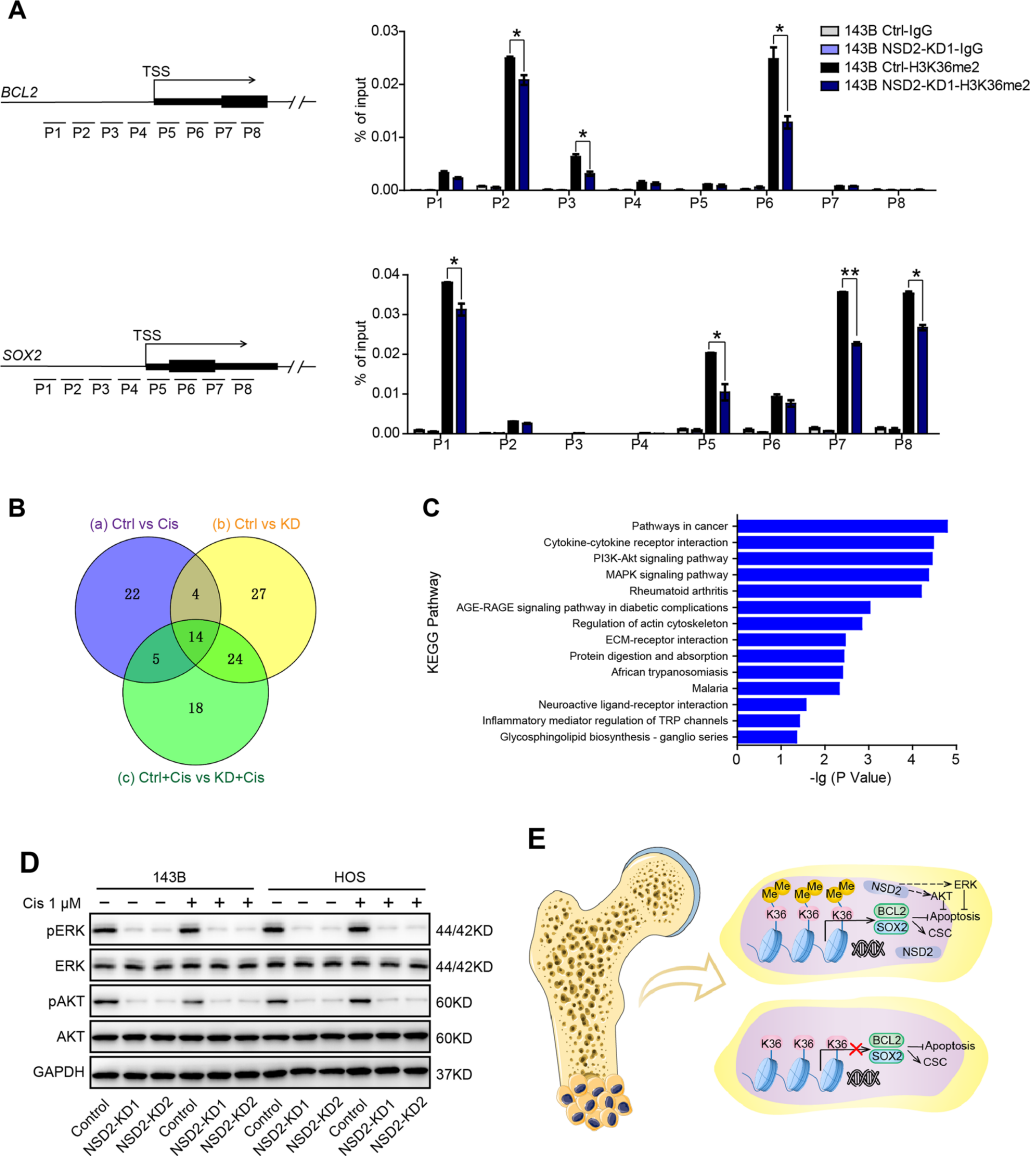
NSD2 mediates H3K36me2 modification at *BCL2* and *SOX2* loci and regulates the extracellular signal–regulated kinase (ERK) and AKT signalling pathways. **a** Chromatin immunoprecipitation–quantitative polymerase chain reaction analysis of H3K36me2 enrichment in the *BCL2* and *SOX2* gene promoters in control and NSD2-KD osteosarcoma (OS) cells. **b** Venn diagram of the enriched pathways between the indicated groups. **c** Fourteen pathways shared by circles a, b and c in **b**. **d** Western blot analysis of GAPDH, AKT, pAKT, ERK and pERK expression in control and NSD2-KD OS cells with or without cisplatin. **e** Schematic diagram of the mechanism by which NSD2 mediates OS genesis and chemosensitivity. NSD2 can directly increase H3K36me2 levels at *BCL2* and *SOX2* loci and activate ERK and AKT signalling pathways to facilitate OS genesis and chemoresistance. *P < 0.05; **P < 0.01

NSD2 is a histone methyltransferase that catalyses H3K36me2, and H3K36 methylation is a histone marker that generally promotes gene transcription. Thus we further investigated whether NSD2 regulates *BCL2* and *SOX2* expression directly by modifying H3K36me2 at their gene loci. The results of ChIP-qPCR revealed that NSD2 knockdown reduced H3K36me2 modification and NSD2 enrichment on the *BCL2* and *SOX2* gene promoters (Fig. 7a and Fig. S3), indicating that knockdown of NSD2 may increase chromatin condensation at *BCL2* and *SOX2* gene loci and thereby blunt the transcription of these two genes in OS cells. Moreover, pathway analysis demonstrated that 45, 69 and 61 pathways were enriched between the control (Ctrl) and cisplatin-treated (Cis) groups (circle a), the Ctrl and NSD2-KD (KD) groups (circle b) and the control with cisplatin (Ctrl+Cis) and NSD2-KD with cisplatin (KD+Cis) groups (circle c), respectively (Fig. 7b). Notably, 14 pathways were shared among circles a, b and c, including the phosphoinositide-3 kinase–AKT pathway and the mitogen-activated protein kinase pathway (Fig. 7c). Western blot analysis illustrated that phosphorylated ERK and phosphorylated AKT were downregulated upon NSD2 knockdown, while the levels of ERK and AKT were not significantly altered (Fig. 7d). Taken together, these results demonstrate that NSD2 directly regulates *BCL2* and *SOX2* expression by mediating H3K36me2 modification at their gene loci and also regulates the ERK and AKT signalling pathways (Fig. 7e).

## Discussion

OS is a highly malignant bone tumour with poor prognosis. The conventional therapeutic treatments for OS rely on surgical resection of the tumour bulk combined with chemotherapy and/or radiotherapy and significantly improve the 5-year survival rate of OS patients up to approximately 60–70%. However, the frequency of recurrence and chemotherapy resistance is high, and these issues are the leading contributors to decreased survival time in patients^43,44^. Thus new strategies need to be introduced into treatment regimens.

Since it is widely acknowledged that epigenetic modulation plays an important role in cancer progression and resistance to chemotherapy, introduction of agents targeting epigenetic changes could be a new anticancer therapeutic strategy to treat the chemoresistance problem in OS^45^. In our study, we first found that NSD2 is upregulated in OS patients, especially in those who are resistant to cisplatin treatment. Additionally, we found that patients with lower NSD2 expression have better prognoses. Then we conducted several experiments in vitro and in vivo, and the results demonstrated that knockdown of NSD2 enhances OS cell apoptosis and has synergistic effects with cisplatin against OS. Lu et al.^46^ likewise reported that NSD2 knockdown could inhibit OS progression, but these researchers did not further investigate the effects of NSD2 on chemosensitivity in OS. In our study, NSD2 knockdown also repressed the expression of genes associated with CSC properties, such as *CD133*, *CD117* and *SOX2*. CD133 (also known as prominin-1) is a cell surface transmembrane glycoprotein that has been used to identify and isolate putative CSCs in several types of solid tumours^40^, and CD117 (KIT) is also a well-known cell surface marker for stem cells^47^. SOX2 has been shown to maintain OS CSCs^48^. Besides, Tang et al.^49^ reported that knockdown of SOX2 inhibited OS cells invasion and migration and Maurizi et al.^50^ also reported that SOX2 was required for OS development and proliferation. Mechanistically, GO analysis revealed several apoptotic and stem cell-related terms that were enriched for differentially expressed genes between the NSD2-KD group and the control group with or without cisplatin treatment. Upon NSD2 knockdown in OS, BCL2 and SOX2 were downregulated, but BAD was upregulated. Pathway analysis further clarified that the ERK and AKT signalling pathways might mediate the process by which NSD2 knockdown enhances OS apoptosis and strengthens cisplatin efficacy.

However, although we revealed direct regulation of BCL2 and SOX2 expression by NSD2, the core subunits or relevant co-factors were not elucidated. Therefore, the underlying mechanisms by which NSD2 recognizes specific genes and modulates their epigenetic modifications remain undefined. In addition, histone methyltransferases can also be involved in methylation of non-histone proteins. For example, the H3K36me3 methyltransferase SETD2 can directly bind to signal transducer and activator of transcription factor 1 (STAT1) and promote its methylation on lysine 525 (K525), leading to STAT1 phosphorylation and activation^51^; however, whether NSD2 mediates modification of certain non-histone proteins to regulate tumourigenesis or chemosensitivity warrants further investigation.

In summary, our evidence of the involvement of NSD2 in OS provides a new perspective regarding possible epigenetic regulation in tumourigenesis and apoptosis as well as in chemosensitivity. The results of this study suggest that NSD2 could be a new therapeutic target in combined chemotherapy to enhance OS apoptosis and the response of OS to chemotherapy. In addition, NSD2 could be a prognostic factor for OS. Thus this study has elucidated new strategies for clinical OS treatment regimens.

## Materials and methods

### Patients and tissue sample collection

The patients with histologically confirmed OS involved in this study were hospitalized at Shanghai Ninth People’s Hospital. All participants signed informed consent documents, and all experiments were conducted according to the guidelines of the ethics committee of Shanghai Ninth People’s Hospital. The clinical information of the patients is summarized in Tables S1 and S2. Based on analysis of cisplatin efficacy, patient biopsies were divided into a cisplatin-sensitive group (inhibition rate 50–100%) and a cisplatin-resistant group (inhibition rate 0–50%). There were no significant differences regarding age or sex between the two groups (*P* > 0.05).

### Immunohistochemistry

IHC was performed on 5-μm-thick formalin-fixed paraffin-embedded slices. After deparaffinization, rehydration, antigen retrieval and endogenous peroxidase inhibition, the samples were incubated with anti-NSD2 (Abcam), anti-TUNEL (Roche) and anti-cleaved caspase 3 (Servicebio) antibodies at 4 °C for 8–10 h. After the secondary antibody was applied, diaminobenzidine (DAKO) solution was used as a chromogen. Furthermore, haematoxylin (Sigma) staining was performed to identify nuclei. Images were acquired using a microscope (Leica DM 4000B).

### Cell lines and cell culture

The human OS cell lines 143B and HOS were purchased from the American Type Culture Collection. The cells were grown in Dulbecco’s modified Eagle’s medium (HyClone) with 10% foetal bovine serum (Gibco, Australia) and antibiotics (100 U/ml penicillin, 100 µg/ml Streptomycin) in a 37 °C humidified atmosphere with 5% CO_2_.

### Cell viability assay

A cell viability assay was performed as previously described^52^. In brief, cells were added into 96-well plates at a density of 5 × 10^3^ cells/well and incubated with or without reagents for 72 h. Then a 1:10 dilution of CCK-8 reagent in culture medium was added into each well, and the cells were cultured for 2 h while protected from light. Then the optical density was read at 450 nm, and data were collected.

### Real-time quantitative polymerase chain reaction

RNA was isolated using TRIzol Reagent (Invitrogen), and 1 μg of each sample was subjected to RT-qPCR using a TaKaRa RT-PCR kit (Takara, Shiga, Japan) following the manufacturer’s protocols. Relative quantification (RQ) was derived from the cycle threshold (Ct) using the equation RQ = 2^−ΔΔCt^. The sequences of the primers are listed in Table S3.

### Western blot analysis

Protein was extracted under the indicated conditions, and the protein lysate was separated by 10% sodium dodecyl sulphate-polyacrylamide gel electrophoresis. The proteins were then transferred to polyvinylidene fluoride membranes. After being blocked in 10% non-fat milk, the membranes were incubated with specific primary and secondary antibodies. The antibodies used were anti-SOX2 (Active Motif), anti-NSD2 (Cell Signaling Technology, CST), anti-H3K36me2 (Active Motif), anti-H3K27me3 (CST), anti-BCL2 (CST), anti-GAPDH (CST), anti-H3 (Abways), anti-ERK (CST), anti-pERK (CST), anti-AKT (CST) and anti-pAKT (CST).

### Colony-formation assay

A colony-formation assay was conducted as previously described^52^. Briefly, 1000 cells were added into 6-well plates, and after cell attachment, different treatments were added. On day 7, the colonies were fixed, stained with 0.1% crystal violet and counted.

### Flow cytometric analysis

The percentage of cells undergoing apoptosis was evaluated by flow cytometry (Beckman Gallios). After incubation with cisplatin for 48 h, the cells were harvested, washed and resuspended in 100 μl of binding buffer with 1 µl of 7-aminoactinomycin D (7-AAD; BD Biosciences) and 5 µl of annexin V (BD Biosciences). To assess cell proliferation, the cells with the indicated treatments for 24 h and subsequent experiments were performed using an EdU Cell Proliferation Kit (Beyotime) as instructed. Briefly, the cells were incubated with 10 mM EdU for 2 h, washed, immobilized and permeabilized. Then the cells were treated with Click Additive Solution for 30 min and stained with 7-AAD.

### Tumoursphere formation

Single-cell suspensions were added to ultralow-attachment plates in defined medium^53^ at a density of 1000 cells/well. Half of the volume of the medium was changed every second day. Images were obtained on day 7 under an inverted microscope. Only spheres >100 μm were quantitated.

### Short hairpin RNAs

Two NSD2-specific shRNAs (sequences listed in Table S4) were designed, and a scramble nonsense sequence was used as a negative control. Lentivirus was packaged using a three-plasmid system including pLVX-shRNA1 with targeted or nonsense sequences, psPAX2 and pMD2G. Stable cell lines were screened with 2 μg/ml puromycin for 2 weeks.

### Mice

Mice were bred and maintained in specific pathogen-free conditions at the animal experimental centre of Shanghai Ninth People’s Hospital. All animal experiments were approved by the ethics committee of Shanghai Ninth People’s Hospital. Twenty-four BALB/c athymic nude mice (6–8-week old) were subcutaneously injected with 1 × 10^6^ of the indicated OS cells. Tumour volume was evaluated every 7 days as 1/2 (a ×b × b), where *a* is the major tumour axis length and *b* is the minor tumour axis length. Tumour weight was measured after the mice were sacrificed on day 21.

### RNAseq and GO analysis

RNA samples were obtained from parental and NSD2-KD1 143B cells that were untreated or treated with cisplatin (1 μM) for 24 h. After the proper pretreatment, the samples were sent to Oebiotech Co. Ltd. for sequencing. Gene expression levels were analysed with the htseq-count^54^ and Cufflinks^55^ programs. All differentially expressed gene lists (*q* value < 0.05) generated by DESeq^56^ were subsequently analysed for enrichment of biological terms with the Database for Annotation, Visualization and Integrated Discovery (DAVID) bioinformatics platform.

### ChIP-qPCR analysis

Control and NSD2-KD1 143B cells (2 × 10^7^ cells) were crosslinked, lysed and sheared using a UCD-300 (Bioruptor) to ~200–700 base pairs in length. ChIP was then performed using an EZ ChIP Kit (Millipore; 17-371) according to the manufacturer’s instructions. ChIP-enriched DNA was then quantified by RT-qPCR. The ChIP antibodies used were anti-H3K36me2 (Active Motif) and anti-NSD2 (Abcam). The primer sequences are listed in Table S3.

### Statistical analysis

All experiments were repeated at least three times as indicated. The mean, standard error of the mean (SEM) and *p* values based on two-tailed *t* tests were calculated with Excel (Microsoft). Differences were considered significant at *P* < 0.05.

## Supplementary information

Fig. S1. H3K27me3 levels in parental and NSD2-KD OS cells as measured by western blot analysis

Fig. S2. Cell cycle progression in parental and NSD2-KD OS cells as analysed by flow cytometry

Fig. S3. NSD2 enrichment at *BCL2* and *SOX2* gene loci in control and NSD2-KD 143B cells as assessed by ChIP-qPCR. *P<0.05; **P<0.01

Supplementary Tables

Supplemental figure legends
