# Supplementary figures and images for: Histone methyltransferase NSD2 regulates apoptosis and chemosensitivity in osteosarcoma
Source: Cell Death Dis. 2019 Jan 25;10(2):65. doi: 10.1038/s41419-019-1347-1 (PMC6347630; doi:10.1038/s41419-019-1347-1)

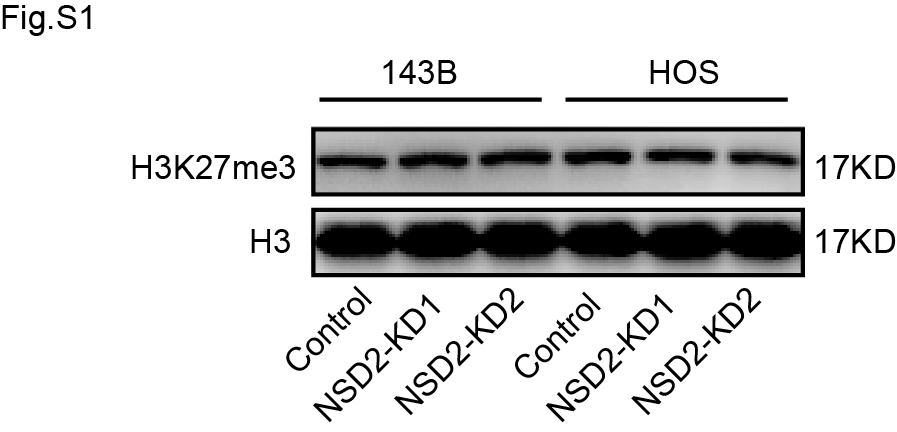

Supplement: Supplementary file 1 — Fig. S1. H3K27me3 levels in parental and NSD2-KD OS cells as measured by western blot analysis [file 41419_2019_1347_MOESM1_ESM.tif]

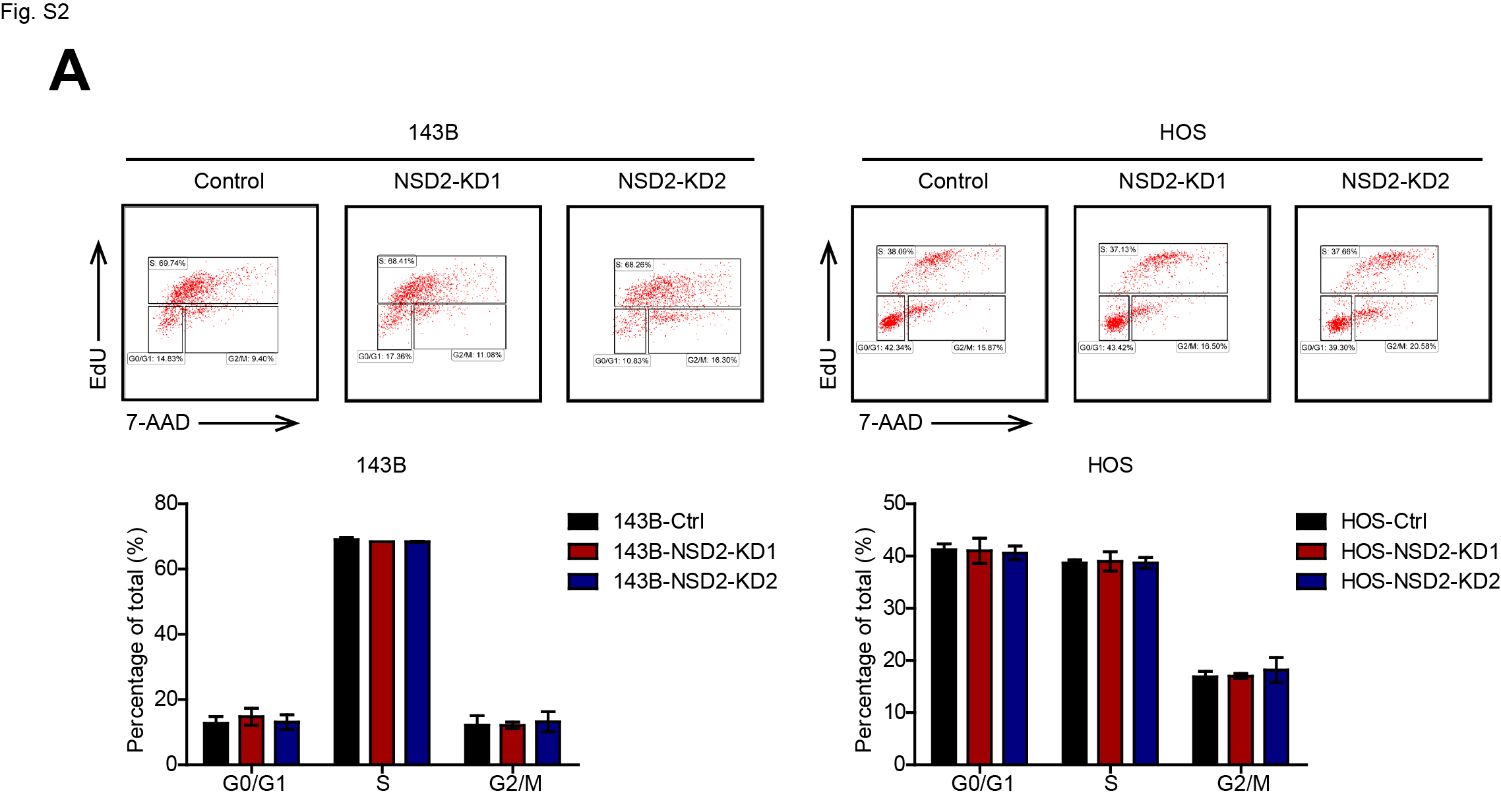

Supplement: Supplementary file 2 — Fig. S2. Cell cycle progression in parental and NSD2-KD OS cells as analysed by flow cytometry [file 41419_2019_1347_MOESM2_ESM.tif]

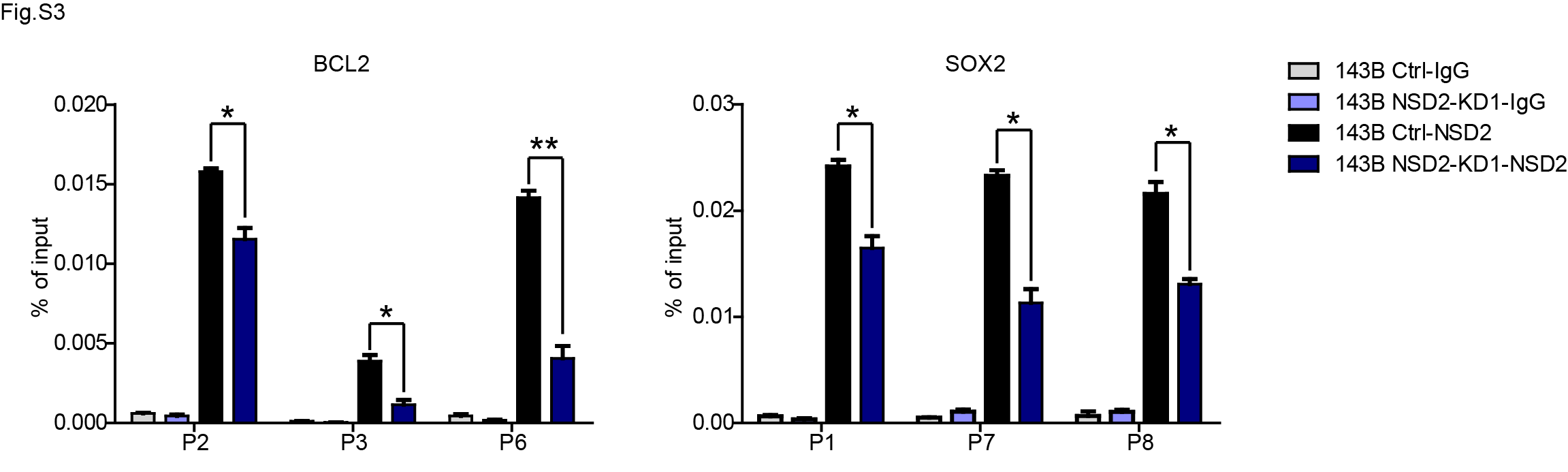

Supplement: Supplementary file 3 — Fig. S3. NSD2 enrichment at BCL2 and SOX2 gene loci in control and NSD2-KD 143B cells as assessed by ChIP-qPCR. *P<0.05; **P<0.01 [file 41419_2019_1347_MOESM3_ESM.tif]
