# Supplementary material for: Histone methyltransferase NSD2 regulates apoptosis and chemosensitivity in osteosarcoma
Source: Cell Death Dis. 2019 Jan 25;10(2):65. doi: 10.1038/s41419-019-1347-1 (PMC6347630; doi:10.1038/s41419-019-1347-1)
Supplement: Supplementary file 4 — Supplementary Tables [file 41419_2019_1347_MOESM4_ESM.doc]

**Supplementary Tables**

**Table S1.** **The** **clinical information of 20 paired osteosarcoma specimens**

| **Variables** | **All patients** |
| --- | --- |
| Numbers | 20 |
| Age at diagnosis, yr. | 10-78 |
| Year of surgery | 2006-2014 |

**Table S2.** **The clinical information of cisplatin sensitive (n=4) and resistant (n=8) specimens**

| **No.** | **Gender** | **Age** | **Pathology** | **Cisplatin Sensitivity** |
| --- | --- | --- | --- | --- |
| 1 | Male | 18 | OS | 54.28% |
| 2 | Male | 48 | OS | 85.58% |
| 3 | Male | 22 | OS | 58.50% |
| 4 | Male | 41 | OS | 83.68% |
| 5 | Female | 26 | OS | 0.00% |
| 6 | Male | 46 | OS | 0.00% |
| 7 | Male | 34 | OS | 9.42% |
| 8 | Male | 15 | OS | 0.00% |
| 9 | Male | 14 | OS | 8.19% |
| 10 | Female | 78 | OS | 0.00% |
| 11 | Male | 17 | OS | 0.00% |
| 12 | Female | 10 | OS | 0.00% |

Cisplatin Sensitivity 50.00-100.00% defined as sensitivity; 0-50.00% defined as resistance. Abbreviation: OS, Osteosarcoma.

**Table S3. Sequences of the RT-qPCR**

| **Gene Name** | **Forward** | **Reverse** |
| --- | --- | --- |
| **NSD2** | CCCACCATACAAGCACAT | TCAGACACTCCGAATCAAA |
| **GAPDH** | TCTGATTTGGTCGTATTGGG | GGAAGATGGTGATGGGATT |
| **BCL2** | GTGGCCTTCTTTGAGTTCG | CATCCCAGCCTCCGTTAT |
| **BAD** | CCCAGAGTTTGAGCCGAGTG | CCCATCCCTTCGTCGTCCT |
| **SOX2** | GCCGAGTGGAAACTTTTGTCG | GGCAGCGTGTACTTATCCTTCT |
| **CD117** | CGTTCTGCTCCTACTGCTTCG | CCCACGCGGACTATTAAGTCT |
| **CD133** | AGTCGGAAACTGGCAGATAGC | GGTAGTGTTGTACTGGGCCAAT |
| **ChIP-BCL2-P1** | TATCTTGGAGGCTGGTGT | TTAGCAGAGCGTAGTGGC |
| **ChIP-BCL2-P2** | GGTCTCCACCTTTGCCTC | CCAATAATCCAGTGTCCCTA |
| **ChIP-BCL2-P3** | CGGACTAGGTGTTCAGGT | CCGTGTATGTGGGAGTGT |
| **ChIP-BCL2-P4** | GGTGCCTGTCCTCTTACTT | AGGAGGGCTCTTTCTTTC |
| **ChIP-BCL2-P5** | CCAGGAGGAGGAGAAAGG | GGATAAATGAAGGCAGGAC |
| **ChIP-BCL2-P6** | TAAGGCAACGATCCCATC | GACTTCTGCGAATACCGG |
| **ChIP-BCL2-P7** | TGTATGCCCTGCTTTCAC | CGGTTATCGTACCCTGTTC |
| **ChIP-BCL2-P8** | CCACAGGGCGATGTTGTC | GAGTGGGATGCGGGAGAT |
| **ChIP-SOX2-P1** | TGACAGTAACAGGCTAGGGA | AGGGCTTAAACCATCAGG |
| **ChIP-SOX2-P2** | AGGCTTTGTTTGACTCCG | ATCCCACGGCACTGTATG |
| **ChIP-SOX2-P3** | AAAGCCGCACGACCGAAAC | GACCCAAACCTCTGTCCTCAAA |
| **ChIP-SOX2-P4** | GAGCGGGAGAACAATGAC | AAGGAAGTGGGTAAACAGC |
| **ChIP-SOX2-P5** | CCGCCGATGATTGTTATT | CAGGAGTTGTCAAGGCAGAG |
| **ChIP-SOX2-P6** | CAACTCGGAGATCAGCAA | GCAGCGTGTACTTATCCTTCT |
| **ChIP-SOX2-P7** | CTCCCATTTCCCTCGTTT | GGTTCGGTGGTCAAGTCC |
| **ChIP-SOX2-P8** | AGAACACCAATCCCATCC | AAGCTCCTACCGTACCACT |

**Table S4. shRNA Sequences**

| **NSD2 shRNA1** | Top strand | gatccG**TGCCAATAACACGTCCACTTTCAAGAGAAGTGGACGTGTTATTGGCA**TTTTTTACGCGTg |
| --- | --- | --- |
|  | Bottom strand | aattcACGCGTAAAAAA**TGCCAATAACACGTCCACTTCTCTTGAAAGTGGACGTGTTATTGGCA**Cg |
| **NSD2 shRNA2** | Top strand | gatccG**CCCTTCGCAGTGTTTGTCTTTCAAGAGAAGACAAACACTGCGAAGGG**TTTTTTACGCGTg |
|  | Bottom strand | aattcACGCGTAAAAAA**CCCTTCGCAGTGTTTGTCTTCTCTTGAAAGACAAACACTGCGAAGGG**Cg |
