# Supplementary material for: Histone methyltransferase NSD2 regulates apoptosis and chemosensitivity in osteosarcoma
Source: Cell Death Dis. 2019 Jan 25;10(2):65. doi: 10.1038/s41419-019-1347-1 (PMC6347630; doi:10.1038/s41419-019-1347-1)
Supplement: Supplementary file 5 — Supplemental figure legends [file 41419_2019_1347_MOESM5_ESM.docx]

**Fig. S1. H3K27me3 levels in parental and NSD2-KD OS cells as measured by western blot analysis.**

**Fig. S2. Cell cycle progression in parental and NSD2-KD OS cells as analysed by flow cytometry.**

**Fig. S3. NSD2 enrichment at *BCL2* and *SOX2* gene loci in control and NSD2-KD 143B cells as assessed by ChIP-qPCR.** *P<0.05; **P<0.01.
